# LLMs in the Loop: A Survey of Language-Driven Driver Monitoring and Assistance Systems

**DOI:** 10.3390/s26092870

**Published:** 2026-05-04

**Authors:** Vanchha Chandrayan, Ignacio Alvarez

**Affiliations:** Human Centered Intelligent Systems, Technische Hochschule Ingolstadt, 85049 Ingolstadt, Germany; ignacio.alvarez@thi.de

**Keywords:** LLM reasoning, Advanced Driver Assistance Systems (ADAS), in-cabin sensing modalities, human–vehicle interaction

## Abstract

In recent years we have seen large language models (LLMs) demonstrating robust reasoning capabilities comparable to human performance. This makes them increasingly appealing for driver assistance, where adaptation to dynamic human context is essential. Yet, research in this area remains fragmented, often focusing on isolated applications, lacking utilization of LLM’s full potential to deliver integrated, context-specific support and action. This survey synthesizes recent advancements in LLM-driven occupant monitoring systems, focusing on their capabilities for interpreting driver states and acting appropriately, enabling a new generation of intelligent driver assistance. We critically examine pioneering frameworks, benchmarks, and foundational datasets that employ techniques like reasoning chains, multimodality, and human-in-the-loop feedback to create personalized and safe driving experiences. We lay out the current trends, limitations, and emerging patterns, in addition to a novel human-centered evaluation of the field, providing researchers with a roadmap towards transparent and trustworthy in-cabin systems that bridge safety with driver experience.

## 1. Introduction

Despite years of progress towards fully autonomous driving, most systems deployed today operate at intermediate levels of automation. At SAE levels 2 and 3 vehicles can manage key vehicle controls such as lane centering or adaptive cruise, but they still depend on human oversight for safety and accountability [1]. This continuing reliance on drivers remains safety-critical. In the United States alone, 3208 people were killed in crashes involving distracted drivers in 2024 [2], while drowsy driving has been estimated to contribute to 17–21% of fatal crashes in 2017–2021 [3]. At the same time, although overall road fatalities have shown some reduction globally and in the EU (3% decrease in fatalities in 2025), road crashes still remain a major public safety burden, and current progress remains insufficient to meet long-term road-safety targets such as the EU’s 2030 reduction goal and Vision Zero ambition [4,5]. Moreover, partial driving automation is becoming increasingly common in consumer vehicles, even though drivers continue to share responsibility for supervision and fallback. Advanced Driver Assistance Systems (ADAS) and increasingly Driver Monitoring Systems (DMS) have become essential components of this technology roadmap, with regulatory mandates in regions like the EU requiring in-cabin monitoring of driver states [1,6]. These systems promise to improve safety by detecting risky driver states and providing corrective support through alerts and warnings. However, the current DMS systems remain limited. They often reduce complex cognitive and affective states to simple labels, fail to generalize across driver populations and environments, and rarely offer explanations or dynamic feedback to the driver [7,8]. As a result, drivers may distrust or ignore them at the moments where assistance is most needed [9].

The emergence of LLMs offers new opportunities to rethink how assistance systems operate. They possess reasoning skills, where they apply complex correlations and causal analysis over the multimodal context, enabling more flexible interpretation and natural-language outputs. These capabilities set LLMs apart from conventional machine learning systems [1]. In various safety-critical domains, such as healthcare and aviation, LLMs are already being tested as human-in-the-loop decision aids [10,11,12]. For in-vehicle context, indicative evidence from reviewed studies suggests that LLM-based approaches offer tangible advantages over conventional methods. For example, vision-language systems for distraction detection have demonstrated zero-shot and few-shot performance competitive with fully supervised baselines [13,14], while retrieval-augmented and memory-enabled assistants have shown improved user acceptance and reduced takeover rates in field experiments [15,16]. Affect-aware conversational agents have been shown to improve perceived system competence and driver compliance compared to pre-scripted alternatives [17,18]. Taken together, these results suggest that LLMs contribute flexibility, contextual reasoning, and interaction quality that rigid pipelines cannot match. These early experiments illustrate the potential of LLM-powered DMS to shift current rigid alert systems towards conversational adaptive assistant agents that act as “co-pilots”.

Still, the path forward is far from straightforward. At the system’s level, today’s assistance technologies face challenges of accuracy, latency, efficiency, and reliability. Models that are too “heavy” (computationally and storage-wise) cannot be supported with real-time requirements on in-vehicle hardware [19]; interventions that are too aggressive may compromise driver autonomy; models that are too opaque may undermine the driver’s trust [20]. At the research level, studies focused on detecting and interpreting driver states remain fragmented as they operate on isolated features, modalities, and evaluation metrics [8]. Reasoning approaches are being proposed, from retrieval-augmented assistance [15] to reasoning chains for risk assessment [13], yet their incorporation into practical in-cabin interactions remains underexplored. Similarly, datasets remain isolated by state or task, limiting opportunities for integrated benchmarking.

**Figure 1:**
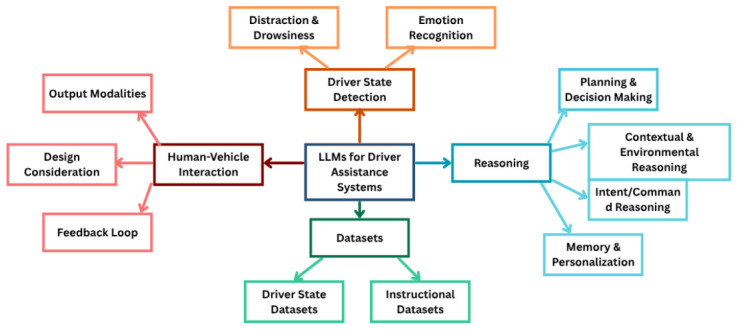
This survey paper focuses on the use of LLMs in the advancement of driver monitoring and assistant systems. The figure shows the scope of this paper.

Our work addresses these gaps by connecting sensing, reasoning, and interaction-based research under real-world vehicular constraints. It is important to situate this survey within the broader landscape of language-driven automotive research. Recent years have seen rapid growth in foundation models and generative AI systems for autonomous driving, including work on embodied driving agents, vision-language-action systems, and broader autonomous-driving foundation-model pipelines [21,22]. While these developments are adjacent and relevant, this survey deliberately focuses on a distinct sub-domain: in-cabin, driver-facing systems that use LLMs and related models to monitor, interpret, and respond to the human driver. External scene understanding, trajectory planning, and vehicle-level control are therefore outside our primary scope, except where they directly inform driver-state reasoning, personalization, or in-cabin interaction design. With this driver-centered scope in mind, we focus specifically on LLM-driven systems that revolve around drivers (Figure 1), asking the following questions:

**RQ1.** How are LLMs used to detect and interpret driver states such as distraction, drowsiness, and emotion?
**RQ2.** How do LLMs reason over multimodal cues, context, and human intent to support decision-making?
**RQ3.** How are in-cabin interactions and interventions enabled by LLMs?
**RQ4.** How do various datasets and benchmarks support or constrain research on LLM-based co-pilots?

The contributions of this paper are as follows. First, we propose a taxonomy of LLM roles in driver assistance, i.e., as detectors, reasoners, and actors. Second, we provide design paradigms emerging across studies, from open-loop warnings to adaptive, conversational co-pilots and identify recurring design tensions. Third, we consolidate datasets across driver states, external context, and language-grounded tasks into a normalized map. Finally, we outline a human-centered roadmap for future intelligent driver assistance systems that integrate transparency, trustworthiness, and inclusivity by design. In doing so, this paper not only surveys a rapidly expanding field but also argues for a shift, i.e., from fragmented detectors towards integrated and adaptive co-pilots that reason *with* drivers, not just about them.

## 2. Background

### 2.1. Traditional Driver Monitoring Systems

Driver monitoring has relied on three primary feature sources, i.e., visual cues (e.g., eyelid closures, yawning, and gaze metrics); physiological measures (e.g., electroencephalography (EEG), heart rate variability (HRV) or respiration rate); and vehicle control behaviors such as steering variability or lane (Table 1) [7]. For instance, drowsiness is often measured through prolonged eye closure or reduced EEG indices [23]; distraction through gaze shifts, head pose, or steering variability [13]; and emotion through facial expression analysis or variations in vocal prosody and HRV [24].

In traditional DMS, these signals are processed through classical machine learning or early deep learning pipelines [8,25]. Facial and ocular features from camera feeds are extracted via Convolutional Neural Networks (CNNs) [26], while physiological indices are classified into driver states using models like Support Vector Machines (SVMs) and decision trees [11,12]. In some cases, temporal patterns are modeled through Recurrent Neural Networks (RNNs) [25]. Training of these systems relies on supervised learning with annotated datasets, often constrained to laboratory environments [27]. Upon detection of a safety-critical driver state, hard-coded interventions were explored to mitigate risky situations (auditory warnings, dashboard icons, or seat vibration) [24]. These approaches provide early safety benefits but come with limitations, including reliance on heavy annotation, rigid categorical states, limited robustness across drivers and contexts, and fixed feedback [8,28].

Another limitation in terms of generalizability is the inconsistency in how these states are defined across studies. Distraction, for instance, may refer to engagement in a secondary task such as phone use [14], gaze deviation from the road scene [8], or reduced situational awareness inferred from steering variability [7]. Drowsiness is similarly variable, measured through eye closure events [29], EEG spectral changes [24], or driving performance degradation [30]. Emotion is the most heterogeneous, ranging from discrete categories (such as anger and fear) [26] and driving-specific affective states (such as stress and boredom) [20,31] to modeling continuous valence-arousal dimensions [18]. Because datasets and models built around these different definitions are often compared as if they measure the same construct, cross-study benchmarking remains unreliable and generalizability is limited. These limitations collectively motivate the move toward more scalable, context-aware, and explainable approaches for next-generation in-cabin monitoring and assistance.

### 2.2. LLMs and Their Optimization

**Figure 2:**
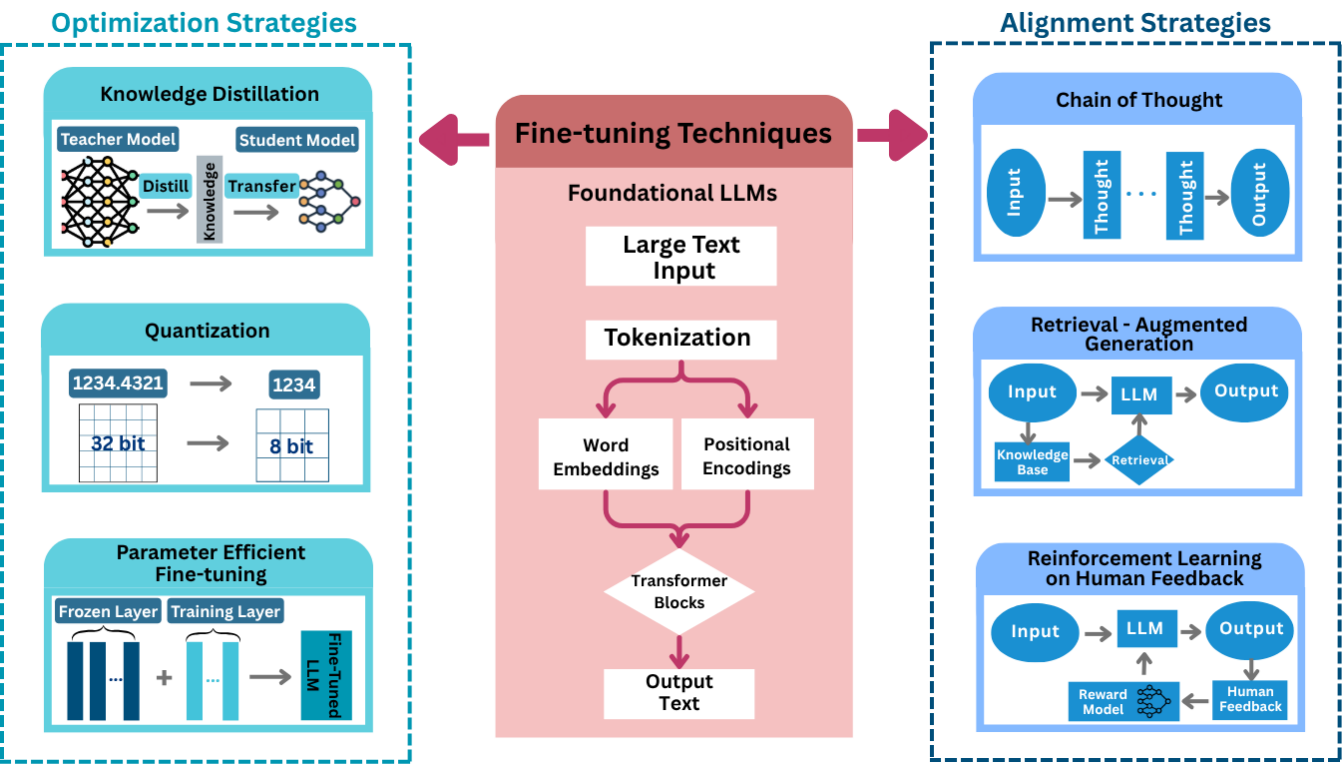
Foundational LLM architecture with fine-tuning and optimization strategies highlighted in the reviewed literature.

The development of LLMs has reshaped how Artificial Intelligence (AI) systems can understand and generate information [1,32]. Built on transformer architecture, LLMs learn patterns across massive text corpora by attending to the relationships between words and sequences (Figure 2) [1]. This training allows them to go beyond simple word prediction. They can support multi-step inference, translate natural language instructions into structured outputs, and adapt to unfamiliar situations with minimal task-specific data [1,33]. These qualities make LLMs particularly appealing for domains where safety and system transparency are central, such as healthcare, aviation, and robotics [10,11,12]. In the vehicle domain, recent surveys note that LLMs have been tested for multiple roles, e.g., supporting route planning and traffic predictions, as conversational assistants for navigation, or explaining outputs and actions of the system [1,32,34]. Such applications show that LLMs can support traditional ADAS by providing a reasoning layer that bridges technical output and driver-facing interpretation. Yet, the same surveys also highlight the limitations of using raw LLMs in real vehicles. Their large size makes them too slow and computational resource-hungry to run reliably on embedded automotive hardware. Their tendency to hallucinate and give nonsensical outputs can undermine system reliability and safety. And their outputs are not automatically aligned with driver preferences, regulatory constraints, and other task-specific requirements [12,19,32]. Therefore, LLMs must be optimized and adapted before they can serve as practical components of driver assistance systems.

Optimization strategies fall broadly into two categories, i.e., making models efficient to run in vehicles and aligning their behavior with the expectations of human users [32]. These approaches are highlighted here as they recur across the frameworks we review, providing a technical foundation for how LLM-based driver assistance systems are being optimized. In an automotive setting, these strategies are closely tied to deployment constraints such as on-device compute, memory, latency, connectivity, and privacy. The following are some techniques widely explored for improving the efficiency of LLMs (Figure 2). Knowledge distillation compresses a larger “teacher” model into small “student” ones that retain performance but operate faster with fewer resources [35,36]. Quantization reduces the precision of stored LLM parameters, thereby shrinking memory utilization and increasing inference speed. Both distillation and quantization approaches enable integration (embedding) of LLM-based systems on vehicle hardware with reduced latency [25,37]. Parameter-efficient fine-tuning (PEFT) techniques like Low-Rank Adaptation (LoRA) and Quantized Low-Rank Adaptation (QLoRA) refine only small portions of the model’s weights, making it possible to personalize models for individual automotive tasks or even individual user support without retraining the full system [13,38] or losing model knowledge/performance.

Alignment strategies focus on ensuring that the model’s outputs are reliable, transparent, and human-centered (Figure 2) [1]. Chain-of-Thought (CoT) prompting encourages models to make their reasoning steps explicit, improving the interpretability of decisions such as why a particular action is unsafe [32,33,39]. Retrieval-Augmented Generation (RAG) grounds LLM outputs in stored knowledge bases such as vehicle manuals, regulations, or real-time weather and traffic databases, thus reducing hallucinations and providing verifiable justifications [15,28]. Reinforcement Learning with Human Feedback (RLHF) tunes model responses to reflect human expectations, ensuring that decisions are not only correct but acceptable to the users [40]. By combining these efficiency and alignment techniques, studies have explored and transformed LLMs from general-purpose models into domain-specific assistant systems that are capable of reasoning in real time, grounding outputs in domain knowledge, and adapting to the needs of their users [1,37].

### 2.3. Vision-Language Models (VLMs) and Multimodal LLMs (MLLMs)

While optimization techniques adapt LLMs for embedded and safety-critical use, another important direction has been extending the reasoning capabilities of these models beyond text. VLMs (such as the CLIP model) jointly learn embeddings (numerical representations of patterns and relationships) from paired images and text. This allows them to connect visual content with natural language descriptions, enabling zero-shot classification (the ability to perform successfully on new visual data from natural language descriptions) and cross-modal retrieval (e.g., searching in text and returning visual data) [8,14,41]. An exploration of VLMs in autonomous driving reports their use in traffic sign recognition, pedestrian detection, and scene captioning, where they provide flexible perception without the need for exhaustive training [8,26,42]. Extending beyond two modalities, MLLMs incorporate diverse streams such as video, audio, telemetry, and sensor data into a unified reasoning framework [1]. Their applications are explored in traffic accident prediction, contextual risk assessment, and integrated perception systems, demonstrating how multimodality improves robustness in dynamic driving environments [1,24].

The trajectory from traditional monitoring pipelines to LLMs and their extension into multimodal systems illustrates the rapid transformation of driver assistance technologies. What was once limited to rigid detection and fixed interventions is now evolving into flexible systems capable of reasoning, adaptation, and interaction. Motivated by this rapid emergence of VLM/MLLM-enabled in-cabin sensing and assistance, we conduct this survey to consolidate scattered evidence into a coherent taxonomy and benchmarking-oriented view, clarifying how language models are being integrated into driver monitoring and assistance pipelines and what system-level trade-offs recur across studies. To support transparency and reproducibility of this survey, the next section details our search strategy, screening procedure, and inclusion criteria used to identify and select the studies reviewed in this paper.

## 3. Methodology

This work presents a narrative review of recent research on language-driven driver monitoring and in-cabin driver assistance systems, with a focus on approaches that integrate LLMs and related VLMs/MLLMs into sensing, reasoning, and interaction pipelines. The scope of the survey covers English-language publications from 2020 onward, reflecting the period in which transformer-based and language-driven models began to meaningfully appear in automotive driver monitoring and assistance contexts. To capture both established and emerging work in this rapidly evolving area, literature searches were conducted using two complementary scholarly databases: Scopus, to identify indexed peer-reviewed publications, and Google Scholar, to broaden coverage to recent conference papers and early-access or preprint work. Searches were organized around thematic keyword classes spanning (i) LLMs and generative AI, (ii) human state interpretation (e.g., distraction, drowsiness, emotion, workload), (iii) reasoning and decision support, (iv) intervention and interaction strategies, (v) multimodal sensing systems, (vi) personalization and adaptation, (vii) explainable AI, and (viii) automotive application contexts such as driver monitoring systems and ADAS. Representative keyword groups and search string patterns used to combine these themes across databases are provided in Appendix A.

**Figure 3:**
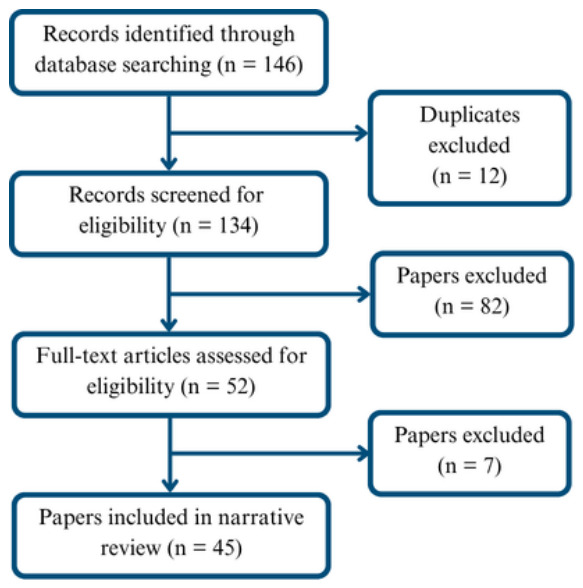
Flowchart of the literature review process.

Across both databases, 146 candidate papers were initially identified (109 from Scopus and 37 from Google Scholar). After deduplication, 12 records were removed. The remaining records were screened in stages using title, abstract, and, when necessary, full-text review. Studies were included if they met all three of the following criteria: (i) the system or framework explicitly employed an LLM, VLM, or closely related language-driven transformer model as a functional component; (ii) the application context was in-cabin driver monitoring or driver-facing assistance (encompassing driver state detection, reasoning over driver context, or driver-directed interaction and intervention); and (iii) the work was available as an English-language publication from 2020 onward. Studies were excluded if they: relied solely on classical machine learning or deep learning pipelines without any language-model component; used LLMs exclusively for external scene understanding, vehicle-level planning, or logistics applications without a driver-facing component; or fell outside the automotive or mobility domain entirely. Dataset papers were retained when they played a substantive role in model development or evaluation within included studies. Following this screening, 89 papers were excluded, resulting in a final set of 45 studies included in the survey (Figure 3). For each included work, key attributes were extracted and synthesized, including the driver state(s) addressed, model modality, the functional role of language models within the system (e.g., detection, reasoning, and interaction), datasets used, and deployment or optimization considerations.

To organize the reviewed literature, this survey uses a three-role taxonomy: *detector-focused studies* (Section 4) are those in which the primary contribution of the language model is state classification or recognition. They identify whether a driver is distracted, drowsy, or in a particular emotional state from sensory inputs. *Reasoner-focused studies* (Section 5) are those in which the language model’s primary contribution is contextual interpretation, planning, or decision support. Thus, going beyond categorical labeling to explain, contextualize, or prioritize information about the driver’s state and environment. *Interaction-focused studies* (Section 6) are those in which the language model’s primary contribution is generating a driver-directed output (a warning, a conversational response, a vehicle action, or an adaptive/closed-loop interaction). These roles are functional and not mutually exclusive. Hence, many systems span more than one category, and studies appearing in multiple sections of this review are placed according to their primary contribution as described in the source manuscript.

## 4. Driver State Detection with LLMs

Although traditional approaches based on handcrafted features and conventional classifiers have significant limitations, they continue to play an important role in today’s systems, particularly for initial feature extraction and for generating supervisory labels. As outlined in the Background section, these pipelines provide the raw inputs upon which newer architectures build. What distinguishes recent research is the exploration of LLMs and VLMs as complementary components: rather than replacing traditional detectors, they extend them by taking on the more complex tasks of reasoning and interpretation [23,25,33]. Given the definitional heterogeneity of driver states discussed in Section 2.1, this survey uses *driver state* as a broad umbrella covering distraction, drowsiness, and emotion; *driver behavior* to refer to observable actions such as gaze direction or secondary task engagement; and *driver condition* to denote broader physiological or contextual factors that may predispose certain states.

### 4.1. In-Cabin Sensing Modalities

**Figure 4:**
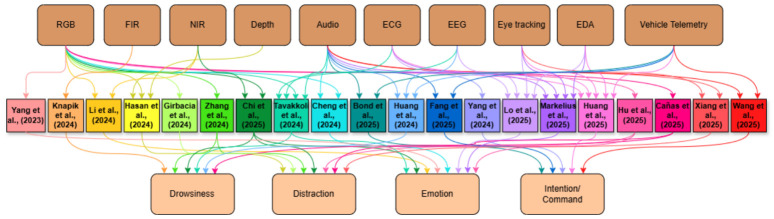
Mapping of representative reviewed studies by sensing modality and target state. References included in order: [7,8,13,14,17,18,20,21,23,24,26,29,30,31,38,41,43,44,45,46].

LLM-based driver assistance systems remain fundamentally grounded in the sensing pipelines that provide access to the driver’s internal states and behaviors. As a result, the choice of sensing modality continues to shape driver states that can be detected, their interpretation, and whether such systems can be deployed in real-time, on-device constraints. Before reviewing state-specific LLM-based detection approaches, we therefore summarize the in-cabin sensing modalities referred to or employed across surveyed literature and highlight their system-level trade-offs. Table 1 synthesizes how different vision-based, physiological, and vehicle telemetry sensors support driver state inference, alongside their key failure modes, privacy implications, and feasibility when integrated into LLM-driven pipelines. Figure 4 complements this overview by visually mapping how different sensing modalities are combined across the reviewed studies for target states.

Across the reviewed literature, several broad patterns emerge in how sensing modalities map onto driver states and system capabilities. Vision-based modalities, particularly RGB and NIR cameras, dominate distraction and emotion detection due to their non-intrusiveness and real-time feasibility. Although they remain sensitive to occlusion and lighting variation [7,8,14,41]. Physiological signals such as EEG and HRV offer higher specificity for latent states like cognitive load and drowsiness, but their intrusiveness and susceptibility to motion artifacts limit deployment readiness [24,31]. Vehicle telemetry (steering variability, pedal dynamics) provides complementary behavioral evidence at low privacy cost and high efficiency, but its indirect and reactive nature means it cannot replace direct driver-state sensing [19,26]. Multimodal combinations (pairing vision with physiological or telemetric streams) consistently improve robustness and coverage across states, as demonstrated by datasets such as AIDE [7] and frameworks such as HSUM [25] and TDSP [43]. However, they introduce additional complexity in fusion, latency, and deployment. These trade-offs reinforce the need for context-sensitive modality selection and highlight that LLM-based reasoning layers must be designed to handle the heterogeneous and sometimes incomplete inputs that real-world sensing pipelines produce.

### 4.2. Distraction and Drowsiness

Distraction and drowsiness have been among the earliest driver states explored using LLMs and VLMs, largely motivated by the limitations of traditional supervised pipelines that rely on heavily annotated data and predefined state categories. Recent work leverages multimodal language-conditioned models to interpret complex driving scenes by jointly processing visual inputs and linguistic context, enabling a more holistic representation of driver behavior and surrounding interactions [8,25]. Hasan et al. introduced DriveCLIP, which adapts CLIP-based vision-language representations to recognize distracted driving actions in naturalistic images and videos [41]. Their framework compares frame-based and video-based variants, showing that temporal modeling substantially improves the recognition of distracting actions. VideoCLIP achieved high Top-1 accuracy on benchmark distraction datasets. Complementing this, Girbacia et al. evaluated multiple open-source VLMs, including PaliGemma, for phone-based distraction detection and showed that limited fine-tuning can yield competitive performance exceeding 95% accuracy [14]. Moving beyond action recognition, Zhang et al. proposed the Distracted Driving Language Model (DDLM), which integrates whole-body pose estimation with a VLM to classify 22 distraction categories and extend recognition toward risk-oriented interpretation [13]. By incorporating reasoning chains, DDLM produces explainable assessments of driver behavior and demonstrates strong zero- and few-shot performance relative to standard baselines. Collectively, these studies illustrate how LLM/VLM-based systems are being used not only for categorical distraction detection but also for temporally aware and explanation-oriented safety assessment.

**Table 1:** In-cabin sensing modalities for LLM-based driver monitoring and assistance.

Sensors	Used for	Key Limitations/Failure Modes	Edge Feasibility	Privacy Risk
**Vision/Audio**				
FIR (thermal) [23]	Drowsiness (yawn, head droop, head pose); distraction	Glasses block eyes; temperature variance; low spatial detail	Med–high efficiency	High
NIR/IR [7,26]	Facial emotion; drowsiness; distraction; robust low-light monitoring	Occlusion (hair, glasses); pose variation; specular reflections	Efficient perception; temporal complexity	Med–High
RGB [7,13,14,41]	Distraction/secondary tasks; facial emotion; drowsiness; body/pose behavior	Low-light sensitivity; occlusion; motion blur; false detections	Low–Med; time-consuming inference	High
Depth/RGB-D [24,29]	Posture/geometry cues; object detection; silent interaction	Noisy depth in real time; reflective surfaces; limited range/placement constraints	Low; 3D processing overhead	Med–High
Audio [17,30,38,44,45]	Emotion; intent/command; fatigue	Background noise; cognitive interference; latency; speech variability	Med–high with hybrid edge-cloud frameworks	Med–High
**Physiological**				
EEG [24,31]	Drowsiness; cognitive state; stress; workload; emotion	Intrusive; noisy; high inter-subject variability	Low; high-dimensional data	High
ECG/HRV [7,20,24,27,29,31]	Arousal; drowsiness; workload	Motion artefacts; signal noise; delayed HRV response	Med; temporal complexity	Med–High
EDA/GSR [18,27,40,43]	Stress; arousal; frustration	Contact sensitivity; drift; influenced by hydration/skin properties/placement	Wearables feasible; ambiguous LLM interpretation	Med
Eye tracking [7,8,17,31,46]	Attention; situational awareness; drowsiness; workload	Lighting variation; occlusion; calibration sensitivity	Med–low efficiency	Med
**Vehicle telemetry**				
Steering wheel (angle/torque/variability) [17,19,24,26,40]	Fatigue; drowsiness; distraction	Reactive (not proactive); indirect correlation; limited personalization	High efficiency	Low
Pedal interaction (brake/throttle) [26,30,47]	Attention level; situational awareness; arousal proxies; driving performance	Context-dependent; confounded by driving style/traffic; one-size-fits-all assumptions	High efficiency	Low

Parallel efforts have focused on improving the robustness and reliability of distraction detection. Hu et al. proposed the Human-Centric Context and Self-Uncertainty-driven MLLM (HSUM), a training-free framework that introduces self-uncertainty estimation and contextual grounding through scene graphs of faces, hands, objects, and environment-specific prompts [25]. In related work, Hu et al. introduced Trustworthy Driver State Perception (TDSP), which combines visual and language features with evidence-based learning to provide calibrated confidence estimates alongside distraction predictions [43]. Both HSUM and TDSP achieve competitive or superior results on public benchmarks such as AIDE and 3MDAD for distraction recognition, with TDSP outperforming DriveCLIP [41]. These studies emphasize uncertainty estimation and calibration as critical system-level requirements for deployment-ready driver monitoring.

A broader survey of VLMs for driver monitoring confirms the strong performance of these models across heterogeneous distraction datasets, while also underscoring persistent challenges related to generalization and evaluation design [8]. Across the literature, in-cabin RGB video remains the dominant sensing modality for distraction detection. At the same time, recent work has begun exploring complementary sensing and multimodal fusion strategies to improve robustness. Knapik et al. demonstrated the use of far-infrared (FIR) thermal imaging to capture distraction-related cues such as head movements and yawning under low-light conditions, positioning FIR sensors as a viable complement to conventional RGB cameras [23]. Other approaches combine visual cues with language-based contextual priors to produce more calibrated and trustworthy predictions [43]. Overall, the distraction literature suggests that video-based perception forms the backbone of current systems, while contextual reasoning and uncertainty modeling are increasingly treated as first-class design objectives.

Drowsiness detection represents another critical safety concern, as fatigue contributes to a substantial proportion of serious road accidents worldwide [30]. Compared to distraction, explicit drowsiness detection using LLMs and VLMs remains relatively underexplored, though early work indicates that similar modeling principles may extend to fatigue-related states. Canas et al. evaluated the open-source VLM Idefics2 on a driver monitoring dataset using zero- and one-shot prompting to detect yawning [8]. While the approach showed some potential for generalization, performance was inconsistent and highly sensitive to prompt design, highlighting the difficulty of modeling subtle and ambiguous states such as drowsiness. Tavakkoli et al. proposed a conceptual framework for multimodal bio-signal fusion that leverages LLM-based contextual reasoning to support holistic fatigue recognition, though the framework was not empirically evaluated [24]. Knapik et al. further demonstrated that far-infrared imaging can robustly capture yawning, head drooping, and eye closure in low-light environments [23]. Several models originally designed for distraction detection, including DDLM [13], HSUM [25], and TDSP [43], also incorporate modules for detecting eye closure or eyelid drooping, underscoring the significant overlap between distraction and drowsiness cues. This overlap suggests opportunities for shared perception components, while also highlighting ambiguity when individual cues may correspond to multiple driver states.

Beyond detection, a smaller body of work explores how LLM-based assistants might intervene once drowsiness is detected, emphasizing the role of adaptive feedback and closed-loop assistance [30,31]. Although primarily focused on intervention, these studies reinforce the importance of reliable and timely detection as a prerequisite for effective driver assistance.

Despite ongoing progress, several challenges remain in achieving robust distraction and drowsiness detection. Real-time monitoring of psychophysiological states is affected by lighting variation, sensor noise, and inter-individual differences [23]. Annotation of distraction and drowsiness remains costly and subjective due to the lack of standardized definitions and ground truth. In addition, privacy and security considerations arise when processing sensitive in-cabin data, alongside technical constraints related to computational load and reliance on cloud-based APIs [13,24]. Across the reviewed literature, these limitations consistently cluster around sensing robustness, labeling subjectivity and dataset comparability, and deployment constraints such as compute, privacy, and connectivity.

### 4.3. Emotion Recognition

Emotion detection plays a critical role in both driving safety and driver acceptance of assistance, shaping vigilance, and compliance with the guidance. Other alarming states, such as driver distraction, could often stem from unsafe emotional states such as anger or anxiety, leading to traffic accidents [26]. However, unlike distraction or drowsiness, emotion is more subjective and context-specific. Therefore, effective detection should integrate multiple cues and reasoning about how they relate in a situation. As the automated driving systems advance towards SAE level 3 or higher, integrating emotion-aware capabilities into driver assistance systems becomes a necessity to also build trust and ensure a personalized user experience [26,48].

In practice, automotive systems infer emotions from complementary modalities. Non-invasive vision remains mostly applied, using facial expressions and audio cues that can capture prosodic shifts linked to arousal or valence [7,26]. Other modalities also include physiological signals (e.g., HRV, skin conductance, EEG) and behavioral measures (e.g., vehicle behaviors, posture) [7,24,26].

LLM/VLM-based detectors have begun to exploit this rich supervision [24]. At the level of facial emotion detection, a study by Li et al. [26] integrated an InternVL vision transformer (an open-source VLM) with Qwen-2 (an LLM), reporting 100% performance on KMU-FED (camera facing driver dataset) and 74.36% on FER2013 (facial emotion recognition dataset). These results outperformed strong conventional CNN baselines. Other limited modality frameworks like Talk2Drive [16] use LLMs to translate natural verbal commands, including emotional cues, into executable vehicle controls, continuously adapting to individual preferences. However, challenges like occlusion, lighting variations and lack of environmental context further emphasize the need for multimodal systems to comprehensively capture the emotional cues with the contextual information. MLLMs can improve emotional recognition and reasoning, even for subtle changes often missed by traditional models [38]. Models like Emotion-LLaMA employ instruction tuning and a combination of encoders to process diverse modalities and align features for in-depth emotional analysis [38]. According to the conceptual framework introduced by Tavakkolli et al. [24], LLMs also offer contextual understanding, allowing them to interpret nuances like sarcasm and implicit meanings and perform sentiment analysis to assess emotional tone from speech. Training-free pipelines such as HSUM [25] and TDSP [43] also suggest that context- and ambiguity-aware systems can enhance reliability in detecting emotions, even without task-specific training. A recent step toward integrated state monitoring is VLM-DM, proposed by Chi et al. [29]. Their approach shows that combining parameter-efficient LoRA-tuned VLMs with traditional vision encoders can unify distraction, drowsiness, and emotion recognition in a single model with competitive accuracy.

Despite these advancements, several significant challenges remain in developing robust emotion detection systems. One of the main limitations is the limited availability of comprehensive, high-quality datasets, including individual and cultural diversity [29]. The black-box nature of many models also raises concerns about their reliability and capability to accurately understand emotional states [27].

In sum, recent systems strive to detect driver states more holistically by conditioning on human-centric context, quantifying uncertainty, and integrating multimodal reasoning.

## 5. LLM Reasoning over Driver Context

**Figure 5:**
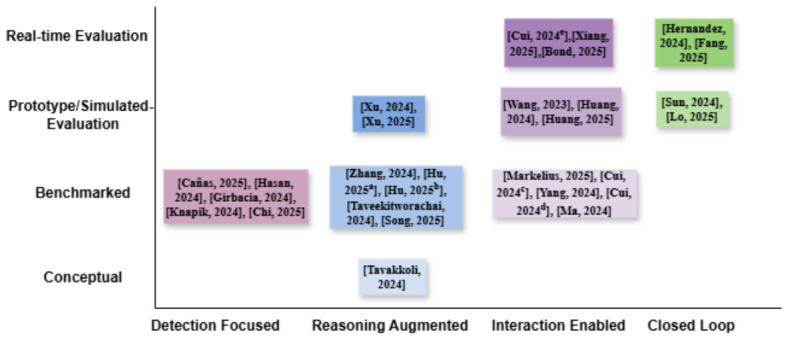
Maturity distribution of representative studies across the functional dimensions used in this survey. The x-axis reflects the degree of functional integration, from detector-focused systems to closed-loop co-pilots. The y-axis reflects the level of evaluation maturity from conceptual proposals to real-time human evaluation. References included in order (bottom to top): Detection focused = [8,14,23,29,41]; Reasoning augmented = [13,24,25] (a), [43] (b), [28,33,36,37]; Interaction enabled = [20,39] (c), [45,49] (d), [16,18,19,30,50] (e), [17,46]; Closed loop = [15,31,40,44].

To synthesize how LLMs are used beyond perception-level processing, we organize prior work along four reasoning dimensions: planning and decision-making, environmental or contextual reasoning, command or intent reasoning, and memory and personalization. These dimensions are intended as analytical lenses rather than mutually exclusive categories, capturing distinct functional roles that language models can play within a driver monitoring and assistance pipeline. Table 2 provides a cluster-level synthesis of the reviewed literature, summarizing recurring methodological patterns, evaluation styles, strengths, and limitations across major functional roles in LLM-based driver assistance systems. Figure 5 complements this synthesis by positioning representative studies according to their functional integration and evaluation maturity, thereby making the distribution of conceptual, benchmarked, prototype-level, and real-time evaluated systems easier to interpret. Because several studies span more than one analytical dimension, they are placed in the figure according to the primary functional focus emphasized in the manuscript.

### 5.1. Planning and Decision-Making

A central capability of LLMs in the driving domain is their ability to act as high-level planners and decision-makers that move beyond rigid rules and statistical controllers. Unlike traditional pipelines, which fail to adapt to a driver’s intent, preferences, or state changes. LLMs bring the capacity to generate, refine, and justify plans that are responsive to both the environment and the human in the loop.

**Table 2:** Cluster-level synthesis of current research in LLM-based driver assistance systems.

Cluster/Functional Role	Typical Model/Method Pattern	Typical Evaluation/Metric Pattern	Recurring Strengths	Recurring Limitations
**Driver state detection & interpretation**	VLM/MLLM monitoring with prompting, reasoning chains, contextual fusion, and occasional LoRA/PEFT; multimodal state modeling is increasingly used [8,13,14,23,24,25,29,31,38].	Mainly benchmark-style recognition metrics (accuracy/F1), with some zero-shot, training-free, or adaptation comparisons; latency is rarely reported.	Richer semantic interpretation of distraction, drowsiness, and emotion; supports uncertainty-aware multi-state monitoring.	Sensitive to overlapping behaviors, ambiguous affect, and fragmented datasets; real-time deployment remains difficult.
**Contextual/environmental reasoning**	LLM/VLM reasoning over driver state, scene, and context using contextual prompting, evidential fusion, multimodal integration, or retrieval grounding [15,24,25,43,44,50].	Mostly offline, scenario-based, or benchmark-style evaluation; reporting is heterogeneous and weakly standardized.	Links state estimates to explanations, risk context, and situational relevance.	Hard to compare across studies; context cues are heterogeneous, ambiguous, or spatially incomplete.
**Command/intent understanding**	LLM/VLM intent decoding, CoT prompting, command grounding, and language-model programs for mapping natural language to structured assistance or control [15,16,19,39,45,46,49,50].	Task-specific intent success, program/policy generation, scenario tests, and selected user/field studies.	More flexible than rigid command pipelines; supports indirect and context-dependent requests.	Phrasing sensitivity, ambiguity, emotional nuance, and safety constraints remain significant.
**Memory & personalization**	Driver profiles, memory modules, RAG, interaction history, and lightweight personalization for continuity and user adaptation [16,19,28,33,37,44,50].	Often scenario-based, qualitative, or small-scale; long-term and cross-driver validation is limited.	Better alignment of warnings, explanations, and dialogue with user preferences and prior interactions.	Tensions with generalization, privacy, storage/retrieval overhead, and cultural bias.
**Adaptive interaction & intervention**	Conversational agents, adaptive warning generation, affect-aware dialogue, and workload-/state-aware interaction strategies [17,18,20,28,30,33,38,46].	More likely to report trust, workload, satisfaction, competence, attention, or physiological outcomes; many remain prototype-level.	More human-centered and context-sensitive assistance; can improve acceptance and perceived competence.	Richer interaction may increase cognitive load or over-reliance; prolonged real-driving validation is limited.
**Closed-loop/integrated co-pilot systems**	Hybrid architectures combining sensing, reasoning, memory, and action in open-, hybrid-, or closed-loop pipelines [15,16,20,24,31,40,44,50].	Mixed evaluation: field studies, prototypes, scenario tests, and conceptual systems coexist; direct metric comparison remains difficult.	Most closely reflects the cognitive co-pilot vision by integrating detection, reasoning, and intervention in one loop.	Computational overhead, latency, privacy, and safety validation remain major barriers; most systems are still prototype-heavy.

In one approach, LLMs are being leveraged as central intelligence for translating complex human instructions and intentions into executable driving actions, making it the decision-making brain of the vehicle. Multiple frameworks such as “Drive As You Speak” [39], LaMPilot [50], and “Receive, Reason, and React” [49] position LLMs as the core decision-maker. Verbal commands from the driver, alongside sensory inputs are interpreted to output language-model programs (LMPs) that can be executed by classical planners. This results in action plans that respect both safety constraints and human intent. Experiments such as “ChatGPT as Vehicle Co-Pilot” [19] also applied this idea by embedding a general LLM into the control loop. This demonstrates that even without extensive fine-tuning, models can adjust trajectories or select appropriate controllers that align with the driver’s preferences expressed in natural language. These frameworks explicitly emphasize LLM’s role in strategic planning, positioning interpretation and planning as two sides of the same reasoning process [50].

Beyond direct command translations, a second cluster of work emphasizes adaptive planning that incorporates driver context and interaction history. The “actor–reasoner” framework introduced by Fang et al. [44] couples a lightweight module that proposes vehicle maneuvers with an LLM-based reasoner that uses CoT prompting to refine these actions in line with human driving styles. Their dual-system approach combined quantitative scenario descriptions with qualitative experience to enhance the adaptability of AV decision-making. Furthermore, LLMs can guide core planning functions using human physiological and behavioral signals. For instance, a framework by Song et al. [37] integrates human behavior and cognitive data (eye tracking and EEG signals) to guide the autonomous driving model’s planning. In parallel, the LLM-enhanced RLHF approach directly integrates LLMs to model human preferences to bias decision policies towards more safe, driver-preferred outcomes [40]. These systems do not simply execute plans but learn how drivers expect decisions to be made, reducing the gap between algorithmic efficiency and human trust.

These developments collectively highlight that by embedding reasoning mechanisms, LLMs enable planning pipelines that make interpretable, reliable, and situationally appropriate decisions. While most frameworks emphasize motion planning, some also extend to decision-making for the design of assistance strategies, thereby determining not only *what* the vehicle does but also *how* it communicates with the driver [20,33]. These approaches are revisited in Section 6. However, the effectiveness of these planning systems depends on how well they interpret the broader driving scene and contextual cues surrounding the driver. The following section examines these environmental and contextual reasoning mechanisms, which provide situational awareness that supports the planning decisions.

### 5.2. Environmental and Contextual Reasoning

Environmental and contextual reasoning refers to how systems go beyond classifying isolated signals to form a narrative understanding of the driver’s state and surroundings [8]. Several recent frameworks demonstrate this shift by integrating information about the traffic environment into LLM reasoning. These systems combine external sensor data, localization, and traffic rules with LLM planning to evaluate road conditions, vehicle distances, and lane availability in real-time [15,46,50]. Similarly, multimodal warning systems adapt their guidance not only to the driver profile but also to situational hazards such as sudden cut-ins or collisions [28]. Collectively, these approaches highlight that assistance quality improves when environmental cues are considered alongside driver state.

In parallel, contextual reasoning also deepens how driver cues themselves are interpreted. As noted earlier in Section 4, systems are increasingly moving beyond a single signal to combine multiple streams (such as body pose, hand movements, voice, and affect) to infer context in richer ways. Approaches like HSUM and TDSP show how adding structured context (scene graphs, descriptive expansions, calibrated confidence) makes detection more interpretable and cautious [25,43]. Another conceptual framework extends this by recommending fusion of vision, speech, and text to align emotional tone with semantic meaning [24]. These works demonstrate that human context is shifting from reactive classification toward proactive interpretation, enabling systems that can explain why a driver might act a certain way, not just what state they are in. However, this rich understanding comes at the cost of greater system complexity, higher computational demands, and new risks of false positives if contextual signals are ambiguous or culturally variable. These challenges underscore the need for another layer of reasoning, i.e., combining information about what drivers do and what surrounds them with what they mean in context when they issue commands or exhibit critical behaviors.

### 5.3. Command and Intent Reasoning

Making decisions and planning actions also rely on a more fundamental capability possessed by LLMs, that is, correctly interpreting what the driver actually means, from explicit directiveness to abstract requests, and even through their emotional tone. Command or intent reasoning addresses this ambiguity by enabling LLMs to infer meaning beyond literal input and ensuring that the assistance systems align with the driver’s underlying goals.

Some studies have framed LLMs as interpreters that translate natural language instructions into structured inputs for the vehicle. Yang et al. [45] demonstrated how LLMs can parse commands into tasks across perception, localization, and control modules, thereby bridging free-form driver input with vehicle functions. Similarly, LaMPilot frames spontaneous user instructions as input for LLMs to generate LMPs [50]. These works highlight the potential of LLMs as intent decoders, but they come with limitations. Benchmarks show that while structured phrasing is handled well by LLMs, ambiguous instructions such as “find a faster way” remain difficult to parse reliably [50]. Such limitations underscore the need for models that can interpret both syntax and human/situational factors simultaneously.

Frameworks like Talk2Drive [16] evaluate LLM-based interpretation of verbal input in field experiments. They demonstrate that the systems must adapt to personal variation in phrasing and the indirectness of commands/requests. By learning from repeated interactions, the model has shown to improve its accuracy to create LMPs even for the indirect expressions like *“I am really in a hurry now”*. Their system has been shown to significantly reduce takeover rate in diverse driving scenarios, indicating enhanced human trust. The Intelligent Driving Assistant System (IDAS) framework expands on this by using RAG to ground driver queries in external knowledge bases like vehicle manuals or other regulatory documents [15]. This reasoning moves beyond "what action to perform" towards “why this request is made”, and it also enables systems to contextualize instructions according to safety criteria or operational restrictions.

Intent is rarely expressed in a neutral form, as emotional states, stress levels, and other affective components greatly alter the meaning of a command. Context-aware frameworks are increasingly exploring how such factors shape interpretation of intent [17]. As explored by Tavakkoli et al. [24], LLMs function as a “cognitive bridge”, integrating emotion and fatigue recognition with intent reasoning. Their framework shows that the same command (e.g., “slow down”) carries different urgency depending on whether it is uttered under stress or casually. Emotion-LLaMA also provides a blueprint for multimodal affect-aware intent reasoning. They propose combining audio, visual, and text encoders to align emotional tone with semantic meaning [38]. These concepts illustrate that intent cannot be separated from *how* it is expressed; hence, integrating affective modulations increases personalization and reliability. Supporting these concepts, a preliminary study introduced an in-vehicle conversational agent, CARA, that uses multi-turn dialogues to make its responses more empathetic and tested it against pre-determined responses [17]. Their approach significantly enhanced perceived competence and trust among drivers. However, these studies also show the risk of reducing robustness when emotional cues are subtle, ambiguous, or culturally varied [17,18,27].

Together, these studies highlight the importance of reasoning over driver commands but also highlight multiple trade-offs. Structured parsing of intent provides precision but struggles with ambiguity, field experiments adapt to variation but rely on limited data, and emotion/affect-aware systems capture the nuances of ambiguous commands but can limit robustness. However, intent is not interpreted in isolation. Humans tend to repeat, refine, or contradict their own instructions over time. To remain effective, another layer of reasoning, utilizing memory and personalization mechanisms, has been explored among the researchers.

### 5.4. Memory and Personalization

As intent reasoning interprets what the driver means in the moment, assistance systems remain limited if they restart the inference process with every interaction [19]. Without continuity, interactions might become repetitive, mechanical, and poorly aligned with driver expectations [16,19]. Memory and personalization mechanisms are being explored to address this limitation. Memory enables the system to accumulate and organize knowledge across interaction and personalization ensures that the system adapts its responses to the same driver over time. Collectively, they move driver assistance from reactive support to adaptive collaboration.

A prominent architectural pattern for achieving this involves integrating a dedicated memory module as a core component of the agent’s framework. This design moves beyond the short context window of a single query to establish an evolving knowledge base [19]. For instance, the LLM-MW system [33] initializes a detailed “individualization profile” for each driver, which is stored in its memory to guide the generation of multimodal warnings. This profile is built and continuously updated by retriving domain-specific knowledge relevant to the driver’s demographic, physical, and experiential characteristics. Other frameworks have further exemplified this approach by designing their system around the memory component. For example, the LLM-PDA framework [28] also constructs personalization profiles that are continuously updated through a dedicated memory system that utilizes MultiQuery-RAG for efficient retrieval. The Talk2Drive framework [16,49] similarly incorporates a memory component to log past interactions and user feedback. Thus, enabling the system to adapt to the driver’s phrasing and preferences over repeated interactions. Another framework that uses a ChatGPT-based driver assistant [19] extends this approach by structuring memory into working, semantic, and episodic layers. This approach offers a more human-like model of remembering past events to support decision-making. The actor–reasoner framework [44] also integrates an interaction memory database; however, in a lighter form. Their memory module retrieves relevant experiences to inform the system to reason about new driving scenarios. These works demonstrate that explicit memory architectures are important to transform assistance into a continuous learning process.

Building on this architectural foundation, the functional application of personalization aims to create a more human-centric and intuitive interaction between the driver and vehicle. Multiple systems achieve this by adapting their behavior to the driver’s immediate cognitive, emotional, and preferential states. The RLHF framework has been used to align vehicle decision policies with human comfort and safety preference [40]. This approach represents the population-level personalization where the system’s behavior is tuned by aggregated feedback. At a more individual level, the D-Twins framework [31] embodies a user’s unique personality and emotional traits by training them on their specific dialogue data, hence enhancing emotional resonance with the user. More lightweight adaptations of personalization are also explored in approaches such as the fine-tuning and quantization framework by Song et al. [37]. Their approach identifies drivers through facial recognition and retrieves stored personalized guidance while remaining resource efficient. Similarly, other studies [7,15,24] also note that psychophysiological, preferential, and environmental cues can also trigger personalization components and are essential for trust and acceptance of autonomous systems.

Ultimately the goal of memory and personalization components is to enable systems that continuously learn and align their decision-making with the user preferences. However, they also introduce new challenges like protecting the driver data, ensuring retrieval efficiency for real-time adaptation, and avoiding cultural biases in personalization aspects. Despite these limitations, such approaches are not just optional enhancements but essential components of LLM-based driver assistance to build more intuitive and trustworthy systems.

## 6. Interaction and Intervention

So far, we demonstrated how LLMs reason to interpret driver states and contextual cues, but their value to the user lies in how assistance is delivered to them. Interaction and intervention form the outward-facing dimension of these systems, where plans and predictions are translated into feedback, guidance, or a dialogue that drivers can perceive and act upon. The emphasis of this section is more about practicality, which outputs and design systems actually work in the cabin.

### 6.1. Output Modalities and Design Considerations

Driver assistance systems rely on three primary channels for feedback—visual, auditory, and haptic [47]. Visual cues are particularly rich sources of information, ranging from dashboard icons and texts to projections on HUDs, but they depend on the driver’s gaze [24]. While auditory (ranging from beeps to spoken commands) and haptic (like steering wheel or seat vibrations) modalities prove effective in capturing attention regardless of visual focus [24,47]. Early systems often relied on a single channel, but research has consistently shown that multimodal feedback outperforms unimodal approaches in reaction times, accuracy, and satisfaction [33].

Recent LLM systems have started to build on this insight by introducing dynamic modality selection. The LLM-based multimodal warning system and LLM-PDA framework [28,33] integrate a planning module that selects what to warn as well as the most effective modality to relay the warning based on contextual factors and driver profiles. Beyond warning and alerts, conversational modalities have also emerged to foster closed-loop interaction mechanisms. Systems like voice-based chatbots, for fatigue mitigation [30], or D-Twins, for boredom mitigation, use a dialogue agent to engage drivers and extend feedback from alerts to ongoing, adaptive interactions. These examples illustrate a shift from static warnings towards more flexible, multimodal interaction strategies where LLMs decide how information is conveyed to maximize salience and minimize disturbance.

Effective interaction design extends beyond selecting modalities; it requires adhering to human-centric principles to ensure interactions are helpful rather than harmful. A primary goal of such interactive systems is managing the driver’s cognitive load and other critical states, as poorly designed or mistimed feedback can become a distraction, inducing stress or even panic [24,28,47]. Following this principle, Xiang et al. proposed an LLM-based persuasion tool that strategically delivers “humanized” persuasive advice, assessing real-time road risks and driver attention to provide load-aware interactions [46]. Other human-centered approaches emphasize that drivers are more likely to accept interventions if they are personalized, empathetic, and transparent [27,48,49]. This was further demonstrated by an empathetic conversational agent proposed by Huang et al. that adapted its interactions based on the induced driver emotional states [18]. Their evaluations against predetermined baselines showed that affective systems led to higher compliance, but conflicting emotions also significantly increased safety-critical scenarios. Researchers are also moving towards design choices that address accessibility issues. For instance, shape coding instead of color coding was proposed for the visual warnings for drivers with color vision deficiency. In the same study, more visual than auditory warnings were provided to foreign drivers who do not understand the local language [33].

These insights highlight that the effectiveness of LLM-based interventions depends on the careful choice of modalities and adherence to human-centric design principles. These principles also help in building driver trust, a prerequisite for the acceptance and adoption of advanced driving technologies.

### 6.2. Open- vs. Closed-Loop

When considering how driver assistance is delivered, another important factor is the system’s underlying adaptivity, ranging from static, predefined responses to real-time, context- and driver state-aware interventions. We divide LLM-based assistance systems into three paradigms based on their interaction loop, i.e., open-loop, hybrid, and closed-loop systems. This spectrum reflects a progression towards increasingly sophisticated and human-centric interactions, where the system’s capacity to perceive, reason, and react to the dynamic user and environmental states dictates the depth of human-AI collaboration.

Open-loop systems deliver interventions based on predefined rules, without adapting to the driver’s real-time state or environmental feedback. In this paradigm, the driver is largely a passive recipient of information, and the system’s responses are fixed. While foundational and low-weight due to their computational simplicity, these systems can sometimes fall short in complex or ambiguous scenarios where adaptation is critical. For example, studies that targeted fatigue mitigation or engaged drivers using multimodal reminders mainly used pre-generated outputs as their interactions. These systems, although effective in their isolated tasks, would have less impact under complex and dynamic real-world changes due to their lack of feedback integration [30,47].

Hybrid systems advance beyond open-loop models by integrating LLM reasoning with elements of feedback and action, though their adaptation is typically partial or discrete rather than continuous. Here, the LLMs function as intelligent intermediaries, performing high-level, human-like reasoning, which then informs a subsequent action or interaction mechanism. There are multiple approaches through which such hybrid interactions are achieved. Command interpretation and action generation systems, where LLMs interpret diverse human intentions, translate them into actionable vehicle controls or interaction strategies [13,16,49]. Another such hybrid system augments LLM reasoning with external knowledge bases or iterative refinement processes to provide context-based aids or personalized responses. Examples of such systems include the IDAS framework that utilizes RAG to ground driver queries in vehicle manuals [15] and the actor–reasoner framework and RLHF loop that performs iterative feedback refinement to bias decision policies towards safer, driver-preferred outcomes [40,44].

**Figure 6:**
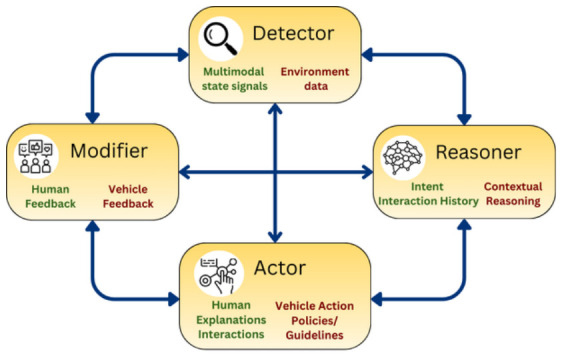
Conceptual closed-loop framework for LLM-based driver assistance.

Closed-loop systems push further towards continuous adaptation, with interventions shaped by real-time monitoring of driver states and contextual cues [46]. D-twins for the boredom framework exemplifies it by detecting boredom in real time through physiological measures and re-engaging the driver with adaptive dialogue [31]. Another study that caters to stress mitigation of drivers altered the length of interaction depending on the real-time monitoring of driver stress [20]. A cross-domain study in aviation also demonstrated the importance of adaptive modification of modality and content of interaction depending upon a pilot’s mental workload, attention, and memory [10]. These systems embody the vision of a cognitive co-pilot, where human states and preferences are continuously integrated in the loop (Figure 6). However, they introduce even higher computational overhead and privacy of sensitive, continuously observed data.

Finally, the effectiveness of driver assistance also depends on whether the driver can understand and trust the system’s reasoning. Current studies already explore different ways to achieve this through interaction design. LLMs enable assistance systems to articulate their decision-making process in natural language, moving beyond “black-box” operations to foster trust between the technology and its users [20,49]. Furthermore, as discussed above, adaptive and personalized transparency through closed-loop interactions is also crucial for enhancing trust and overall driving experience. For example, short and concise feedback is less stressful in manual driving, whereas longer, more detailed explanations can enhance feelings of safety in AVs by increasing the understanding of the system’s decision-making [20]. The concept of bidirectional transparency, where the system not only explains its actions but also shows that it understands the user inputs and intent, also significantly increases user trust, especially in complex and safety-critical scenarios [51]. Collectively, these interactive explainability mechanisms are important for ensuring that LLM-driven assistance is not just functional but also comprehensible, accepted, and genuinely trusted by its users.

## 7. Datasets and Benchmarks

Bridging the gap between perceptive assistance and intuitive interaction, the development of LLM-driven in-cabin systems critically relies on comprehensive and diverse datasets. These datasets provide the ground truth for training models that can understand, reason, and adapt to the complexities of human behavior and dynamic driving environments. This section reviews the key datasets and benchmarks that underpin the advancements of LLM-based driver monitoring and interaction (Table 3).

The first category comprises datasets for driver state detection. Widely used distraction corpora such as StateFarm [52], SynDD1 [53], DMD [54], and NTHU-DDD [55] have enabled recognition of secondary tasks and are still benchmarks for vision-language approach [29,41]. More advanced datasets, like AIDE [7] and 3MDAD [56], extend this scope to include multi-view and multimodal streams for distraction, drowsiness, and emotion [43]. Specialized datasets expand the modality space, e.g., thermal datasets (TFW [57], SF-TL54 [58]) for robust monitoring under low-lighting conditions [23]. For emotion detection, some studies use general datasets like KMU-FED and FER2013 or AIDE for five basic emotions in driving context [26,38]. However, for more sophisticated emotional reasoning, datasets like MERR and CA-MER [59] offer extensive fine-grained multimodal annotations (visual, audio, text) [26]. These datasets illustrate progress but also highlight fragmentation as each focuses on a particular driver state, underscoring the need to build integrated resources that span multiple states and affect naturalistic conditions. Across these resources, the recurring limitations are fragmented state coverage, uneven ecological realism, limited multimodal and language-grounded integration, and weak comparability across annotation schemes, evaluation settings, and user populations.

A second category consists of instruction datasets and benchmarks, which enable LLM systems to evolve into cognitive co-pilots by understanding natural language commands. Language-augmented datasets like Talk2Car [39], nuScenes-QA [60], and DriveLM [61] enrich existing autonomous driving data with natural language commands and visual question answering, facilitating the interpretation of human intent [39,50]. BDD-X further contributes with textual explanations for a vehicle’s self-driving actions, enhancing transparency [32]. To evaluate end-to-end instruction following, benchmarks like LaMPilot-Bench and UCU Dataset quantitatively assess LLM’s ability to reason and classify system requirements based on driver’s demands, from safety instructions to comfort requests [45,50]. These datasets are crucial for developing LLM-driven systems that can monitor driver states, infer and interpret, and translate human intent to personalized and contextually appropriate actions. Yet, they remain limited in scale and diversity, reinforcing the need for integrated benchmarks for cohesive evaluation of intelligent driver assistance systems.

Finally, a notable gap lies in datasets that integrate external driving context with in-cabin states. While general autonomous driving datasets like KITTI [62], nuScenes [63], and BDD100K [64] dominate perception research in tasks such as 3D object detection and motion planning, the driver assistance systems rarely employ them directly. It has been widely demonstrated that external scene factors such as traffic density, weather, or lighting conditions strongly influence driver states, including stress, cognitive load, and distraction [15,28,50]. Therefore, bridging these datasets with in-cabin corpora would allow copilots to align driver state recognition with environmental triggers, enabling more timely, contextually grounded, and empathetic interventions.

## 8. Discussion

This review highlights how the integration of LLMs into driver assistance has moved from fragmented state detectors towards reasoning-driven, adaptive, and interactive copilots. Yet the opportunities and tensions that emerge extend beyond technical improvements; they redefine the interaction paradigm between humans and vehicles. In this discussion, we analyze these tensions across four threads: first, the paradigm shift from traditional ADAS to cognitive co-pilots; second, critical design tradeoffs in human-centered systems; third, rethinking trust in driver assistance; and finally, broader implications for research and ethics.

### 8.1. From ADAS to Cognitive Co-Pilots

Driver monitoring and assistance systems have traditionally been built around modular ADAS pipelines that perceive signals and interpret them into predefined categories. While this architecture has yielded some progress, our review shows it remains fragmented. For instance, only a handful of studies integrate detection and reasoning about context, and interventions are mostly delivered without considering how drivers will make sense of them.

However, studies show that LLMs and MLLMs are capable of enabling a more integrated and human-centric approach. Systems are emerging that utilize LLMs as the central decision-making brains, enabling a loop from perception to feedback adaptation (Figure 6). This involves integrating detection, reasoning over human and environmental context, decision-making, human-machine interaction, and continuous learning into unified frameworks. So far, at the state interpretation level, works like VLM-DM [29], DriveCLIP [41] and DDLM [13] demonstrate that VLMs can generalize multiple states across datasets with minimal fine-tuning. DDLM further showed strong zero- and few-shot performance relative to fully supervised classifiers. At the reasoning level, LLMs and VLMs are being introduced to deliver contextual prompts, reasoning chains, and uncertainty calibrations to connect raw signals with explanations and risk assessments [13,25,43]. At the action level, frameworks increasingly embed reasoning into interventions, parsing vague or affective commands or tailoring warnings to driver profiles. For instance, Talk2Drive [16] reports a significant reduction in takeover rate compared to non-LLM controls, while CARA [17] shows improved perceived competence and trust against scripted-response baselines. Some pipelines also close the interaction loop by providing human feedback to enhance system learning [16,20,31,49]. These dimensions, where conventional ML offers little by design, LLM-based systems provide structured and reasoned natural-language outputs that rigid pipelines cannot match [20,28,33]. Taken together, these results suggest that LLMs contribute meaningfully across detection accuracy, interaction quality, and explainability. However, the evidence base for rigorous cross-paradigm comparison remains limited by the field’s heterogeneous tasks and inconsistent metric reporting.

Despite this progress, most systems fall short of integrating all four dimensions within a single deployable pipeline. Many excel at interaction design but lack robust contextual reasoning, while others demonstrate advanced reasoning without tight coupling to real-time perception. These limitations are primarily driven by computational complexity and latency constraints. Due to their scale, LLMs demand substantial compute resources and often incur nontrivial inference delays. Multimodal architectures further increase memory and processing requirements, particularly when continuous learning or memory components are included, limiting feasibility for embedded in-cabin deployment. Addressing these constraints is therefore central to advancing integrated driver assistance systems.

**Figure 7:**
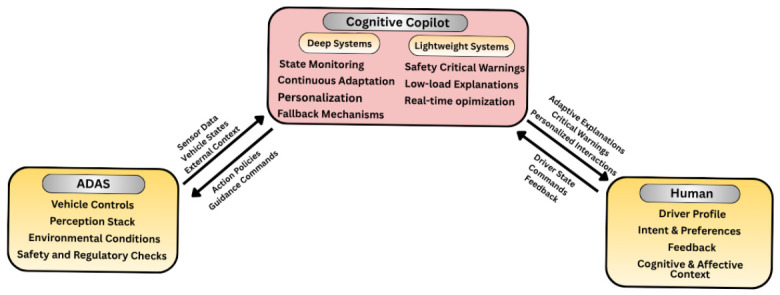
Design recommendations for future driver assistance systems.

A promising direction lies in adopting a “train heavy, deploy light” paradigm, in which resource-intensive multimodal reasoning is leveraged during development and distilled into compact models suitable for in-vehicle execution (Figure 7). This can be achieved through techniques such as knowledge distillation, quantization, and edge deployment. For example, quantized LLMs such as PHI-3 have demonstrated improved inference efficiency in pilot assistance systems [10], while smaller VLMs like Idefics2 are increasingly favored for their computational efficiency [8]. Memory optimization is also critical for reducing system overhead and supporting long-term adaptation, with approaches including selective retention, driver profiling, and lightweight memory modules [19,33]. However, open challenges remain in designing efficient retention strategies and privacy-preserving mechanisms that enable continuous learning without excessive storage or data exposure. Moving beyond siloed state detection, future systems must also integrate multiple safety-critical driver states and affective factors within unified reasoning and action pipelines. This requires leveraging LLMs to interpret rich multimodal inputs, contextualize them with external factors, and translate system inferences into timely, deployable responses [24].

### 8.2. Design Tensions in Human-Centric Driver Assistance

The development of LLM-based driver assistance systems is not only a technical challenge but also a design one. These tensions highlight that effective systems cannot be optimized on a single axis, i.e., personalization, transparency, or efficiency, without carefully considering their trade-offs. Below, we discuss three recurring tensions and outline potential directions to address them.

#### 8.2.1. Personalization vs. Generalization

Several systems emphasize personalization by creating driver profiles, memory modules, or behavior and cognitive models that adapt interactions to individual characteristics and preferences [16,28,31]. While these approaches show promise in improving user acceptance and reducing false positives and unnecessary interactions, they risk overfitting idiosyncrasies that may not translate across various driver profiles, cultures, or driving contexts. In contrast, training-free or evidential-fusion models demonstrate greater robustness across multiple datasets but remain limited in catering to individual needs [25,43]. This tension highlights the lack of evaluation frameworks that test both personalization and generalization simultaneously. Moving forward, frameworks that employ shadow learning and contextual personalization, where systems learn and adapt for certain drives and then deploy, can be studied (Figure 7). Such systems could maintain general safety actions and let phrasing, timing, and modality be according to individual preferences. Additionally, validation studies could utilize split-by-driver or split-by-demographics protocols to report what transfers and what does not [28,49].

#### 8.2.2. Transparency vs. Cognitive Load

Explainability of a system’s planning and decisions is increasingly viewed as an important factor to foster driver trust. However, studies also highlight that more explanation does not always equate to better outcomes. Specifically, in high-risk or stressful situations, shorter feedback is considered more efficient and non-intrusive as compared to detailed explanations [20,30]. On the other hand, autonomous systems with higher agency, when provided with detailed descriptions, lead to fewer takeovers and higher satisfaction [20]. The key challenge is not the absence of explainability but the lack of workload-informed mechanisms for when and how to provide it [46]. A potential solution is to reconceptualize co-pilots as independent agents that not only track the driver’s state but also maintain awareness of the vehicle’s operational design domain (ODD) (Figure 7) [15,50]. By combining the two streams of information, these systems can mediate between the driver and ADAS, delivering explanations that are both contextually grounded and do not risk information overload. This framing could support more adaptive explanation strategies, such as tailoring modality, verbosity, and timing, based on real-time workload assessments. Explanations could also be provided in multiple stages, i.e., brief instruction or feedback during high-workload situations and more detailed explanations on demand.

#### 8.2.3. Efficiency vs. Reliability

As previously discussed, computational costs and latency issues have motivated researchers to develop more efficient and lightweight systems that rely on quantization or knowledge distillation [10]. Although these systems improve on technical integration challenges, they can compromise the subtlety and reliability of state detection and action strategies. For instance, VLM-DM [29] demonstrates that LoRA can unify multiple state interpretations with competitive accuracy but still struggles to reliably reason over overlapping features (reaching behind or dropping down). To mitigate this tradeoff, reliability must be treated as a primary criterion, with efficiency serving as a constraint rather than a goal. Approaches such as “train heavy, deploy light” offer a way forward (Figure 7). Reliability can also be enhanced by integrating fallback mechanisms, such as escalating uncertain or ambiguous cases to slower but more robust models [44]. Adding modality redundancy, like using thermal vision under low lighting conditions or incorporating various state cues from multiple modalities, can also mitigate failures in specific cases [23,24].

### 8.3. Rethinking Trust

Trust in ADAS has traditionally been tied to the predictability and reliability of the system; if the system works smoothly, without interruptions, drivers could trust it. However, with autonomous systems working on higher agency and LLMs supporting with higher reasoning tasks, this conception is no longer sufficient. Trust now hinges on whether the systems can articulate their reasoning, account for uncertainty, and demonstrate self-awareness in their outputs. Frameworks that quantify uncertainty or provide stepwise reasoning explanations have begun to shift trust from binary correctness toward calibrated confidence [13,25,43]. At the same time, state-aware explanations offer a way to manage cognitive load and overwhelming interactions [10,18,46]. Beyond transparency, trust is also shaped by whether the drivers feel the system “knows” them and adapts to their characteristics. Personalization strategies show promise in strengthening the bond between drivers and vehicles, however, with critical caveats. As previously discussed, highly personalized systems raise tensions in scalability, privacy, and cultural diversity since over-standardized empathy can feel manipulative without personalizing on the individual level [16,19,31]. Accessibility further complicates this picture: multimodal warnings may foster trust in general, but if visual cues rely solely on color or auditory responses overlook sensory overload, trust will erode for users with different cognitive and perceptual needs. Few studies have adopted approaches to cater to users with color-vision deficiency or balancing between modalities to achieve inclusivity [28,33]. However, there remains a significant gap in addressing accessibility requirements for a broader spectrum of users, including those with age-related changes, sensory and motor impairments, neuro-diverse conditions (e.g., ADHD, autism), and psychological or affective challenges such as heightened anxiety or stress.

Future works in intelligent driver assistance systems must reframe trust as a design goal that integrates adaptive explainability, context personalization, and accessibility by default. Promising directions include maintaining driver profiles by shadow learning and continuous feedback integration, explain-on-demand mechanisms that balance trust with overwhelming interactions, and adaptive monitoring of safety-critical and pathological states in order to deliver human-like responses and interventions. Equally, the traditional reliability-dependent trust must be safeguarded by constantly improving vehicle actions and incorporating fail-safe mechanisms to mimic human driving behaviors. As ultimately, the credibility of cognitive co-pilots will rest on both perfect predictability and decision-making, and how transparent, empathetic, and inclusive they behave with human drivers.

Figure 7 summarizes our design recommendations for future research on LLM-based driver assistance. We propose conceptualizing the cognitive co-pilot as an agent distinct from the ADAS, mediating between vehicle systems and the human driver. The co-pilot integrates both heavy (deep, fallback) and lightweight (fast, low-load) subsystems, enabling adaptive reasoning, context-sensitive explanations, and reliable actions. By separating this reasoning layer from ADAS control, the architecture supports human-centric collaboration while balancing efficiency, transparency, and trust.

### 8.4. Datasets, Methods, and Ethical Considerations

Another hurdle in the progress towards cognitive co-pilots is the infrastructure that supports their development and deployment. Existing datasets remain fragmented, with some focusing on task-specific driver states, others on traffic scenes, and a very small subset on language-grounded instructions. With only a few benchmarks integrating multimodality sensing with human-centric instructions, this lack of cohesion hinders comparability and evaluation across diverse datasets and frameworks. A related limitation is that current resources also provide only limited coverage of rare or high-risk corner cases, such as extreme weather, sudden road events, or other atypical driving conditions that may strongly shape driver state and assistance needs. Future work must prioritize integrated benchmarks that combine multiple driver state signals with contextual reasoning and natural-language annotations. At the same time, LLMs can support data augmentation, for example by generating synthetic corner cases or complex scenarios that reflect real driving conditions. This is an important future direction for HCI, as the human-partnership capabilities of new LLMs call for benchmarks that are human-centric by design.

Methodologically, most works continue to rely on dataset-bound metrics for evaluation with limited ecological testing. As shown in the reviewed studies, models perform strongly on benchmarks but falter in real-life or simulated testing with human participants. There is a growing need for hybrid methodologies where scalability of automated benchmarks can be combined with the depth of human-centered user studies. Evaluations should extend beyond accuracy checks to include results central to HCI, such as explanation quality, acceptance, and trust.

Finally, the integration of a driver’s sensitive data, e.g., faces, voices, and other physiological signals, raises critical ethical concerns. Ensuring privacy-preserving machine learning, for instance, on-device inference and federated learning, are essential for such systems. Additionally, systems should also account for transparent data collection practices with explicit user agency in deciding what is shared.

## 9. Conclusions

This survey reviewed how LLMs are being integrated into driver monitoring and assistance, moving the field from rigid detectors toward reasoning-driven and interactive systems. The four research questions proposed in the Introduction were addressed through the main synthesis sections of the paper. RQ1, concerning the use of LLMs for driver state detection and interpretation, was addressed in Section 4, where we reviewed multimodal sensing pipelines and the role of language-enabled models in recognizing distraction, drowsiness, emotion, and related driver states. RQ2, concerning the role of LLMs in reasoning over driver context, was addressed in Section 5, which examined contextual reasoning, intent understanding, memory, and personalization. RQ3, concerning how these systems support interaction and intervention, was addressed in Section 6 through the analysis of interaction paradigms ranging from open-loop assistance to more integrated co-pilot systems. RQ4, concerning the datasets and benchmarks that currently support this area, was addressed in Section 7, where we discussed the fragmentation, limitations, and future needs of the available data infrastructure.

Our contribution lies in framing these developments through an HCI lens, providing design recommendations (Figure 7), and emphasizing the need for research infrastructure that reflects human partnership rather than narrow classification. In reviewing this literature, a key challenge was that many of the systems remain prototypes or proofs of concept, with findings often constrained to limited datasets or simulation. A further limitation of the current literature is that many studies report algorithmic improvements without sufficient system-level detail on latency, computational cost, hardware requirements, or deployment trade-offs. Together with the lack of common cross-industry benchmarking protocols, this makes rigorous and unbiased comparison across methods difficult. At the same time, the field is advancing rapidly across all stages of the driver-assistance loop. We therefore position the taxonomy proposed in this survey as an initial organizing framework that can be refined and expanded as future work and can integrate emerging methods for sensing, reasoning, and interaction. Future progress will hence depend not only on better models but also on more standardized, open, and system-oriented evaluation practices. Looking forward, we identify several other directions for future work, including the development of multimodal and multi-agent systems that can close the loop of human–vehicle interaction, integrated benchmarks that combine driver state with contextual and natural language annotations, and more real-world evaluations to assess the usability of such systems. Although the future directions discussed here highlight promising opportunities for language-driven driver assistance, their practical deployment will depend on constraints that are still only weakly addressed in current work. These directions should therefore be understood as research-oriented design trajectories for intelligent driver assistance systems. Overall, this survey argues for a closer integration between sensing technologies, language-based reasoning, and human-centered evaluation to support the development of transparent, trustworthy, and deployable driver assistance systems.

## Figures and Tables

**Table 3:** Datasets used in reviewed studies, with their focus, modalities, and key limitations.

Type	Focus	Datasets	Modalities	Utilization in Reviewed Studies	Key Limitations
**Driver State**	Distraction	StateFarm SynDD1, DMD SAM-DD	RGB RGB RGB	[14,41] [41] [29,41]	Narrow RGB setups; limited multimodal and contextual coverage
	Drowsiness	NTHU-DDD	RGB	[29]	Single-state focus; limited variability and ecological realism
	Emotion	KMU-FED FER2013 MERR, CA-MER	RGB RGB Multimodal (Audio/Visual/Text)	[26,29] [26] [27]	Weak driving specificity; annotation ambiguity; limited naturalistic coverage
	Multi-state	AIDE (Emotion, Drowsiness, Distraction) 3MDAD (Distraction, Drowsiness) TFW/SF-TL54 (Distraction, Drowsiness)	RGB (internal & external view) RGB Far-infrared spectrum	[25,43] [25,43] [23]	Still fragmented across states/modalities; limited language grounding and broad scenario coverage
**Instruction**	Command grounding	UCU Talk2Car	Text RGB + LiDAR + RADAR + GPS + Text (commands)	[45] [39]	Limited scale and user diversity; weak in-cabin state integration
	Reasoning/QA	DriveLM, nuScenes-QA	RGB + LiDAR + RADAR + GPS + textual Q/A	[15]	Strong external-scene grounding but limited driver-state coupling
	Program/Policy synthesis	LaMPilot-Bench	Text (commands) + simulation environment	[50]	Simulation-heavy; limited real-driver or real-time validation

## Data Availability

No new data were created or analyzed in this study. Data sharing is not applicable to this article.

## Appendix A. Literature Search Keywords and Search Strings

Table A1 summarizes the thematic keyword classes used during the literature search, and Table A2 provides representative search string patterns and example queries. The search process was iterative, and keyword combinations were refined until additional searches no longer yielded substantively new eligible studies.

**Table A1:** Representative keyword classes used to structure the literature search.

Keyword Class	Representative Keywords
LLMs and Generative AI	large language model; LLM; GPT; transformer model; generative AI; conversational AI; foundation model; multimodal LLM; vision-language model; language-driven system
Human State Interpretation	human state monitoring; driver state interpretation; cognitive state recognition; affective computing; physiological state estimation; drowsiness; stress; cognitive overload; distraction; fatigue; emotion; trust; situational awareness; attention; workload; mental state; vigilance
Reasoning and Decision Support	reasoning chain; cognitive reasoning; decision support system; common sense reasoning; context-aware decision-making; deductive reasoning; knowledge-grounded AI; causal reasoning; human-like inference
Intervention and Interaction Strategies	intervention system; adaptive intervention; in-vehicle assistant; driver feedback; behavioral feedback; cognitive feedback; conversational AI; multimodal intervention; proactive assistance; in-vehicle interface; verbal intervention; system-initiated alerts
Multimodal Systems and Sensing	multimodal system; sensor fusion; biosignal fusion; visual-language fusion; EEG; EDA; PPG; ECG; facial expression analysis; speech emotion recognition; gaze estimation; fusion-based detection; multimodal feedback
Personalization and Adaptation	personalized assistance; adaptive interface; user-specific adaptation; context personalization; driver profiling; human-in-the-loop; individual traits modeling; context-aware personalization
Explainability and Transparency	explainable AI; transparent decision-making; interpretable model; explainable driver assistant; natural language explanation; user-understandable output; cognitive transparency
Automotive and Driver-Assistance Context	advanced driver assistance system; ADAS; driver monitoring system; DMS; in-vehicle AI; human–vehicle interaction; automotive AI; in-cabin monitoring; driving assistant; intelligent transportation system; semi-autonomous driving; cognitive-aware ADAS

**Table A2:** Representative search string patterns and example queries used across Scopus and Google Scholar.

Search String Pattern	Example Query
{LLM/generative AI term} + {driver monitoring/human state term}	("large language model" OR LLM OR GPT OR "generative AI" OR "vision-language model") AND ("driver monitoring" OR "human state monitoring" OR "driver state interpretation" OR distraction OR drowsiness OR fatigue OR stress OR emotion)
{LLM/transformer term} + {automotive/driver assistance term}	("large language model" OR LLM OR GPT OR "transformer model" OR "foundation model") AND (vehicle OR driver OR automotive OR "advanced driver assistance system" OR ADAS OR "driver monitoring system" OR DMS)
{LLM term} + {automotive term} + {specific driver state/cognitive factor}	("large language model" OR LLM OR GPT) AND (driver OR vehicle OR automotive OR "autonomous driving") AND (stress OR drowsiness OR fatigue OR distraction OR workload OR attention OR "situational awareness")
{LLM/multimodal term} + {sensing/multimodal term} + {driver state term}	("multimodal LLM" OR "vision-language model" OR LLM) AND ("sensor fusion" OR "biosignal fusion" OR EEG OR EDA OR ECG OR PPG OR gaze OR "facial expression") AND ("driver state" OR emotion OR drowsiness OR fatigue OR distraction)
{LLM term} + {interaction/intervention term} + {in-vehicle context}	("large language model" OR LLM OR GPT OR "conversational AI") AND ("in-vehicle assistant" OR "driver feedback" OR intervention OR "verbal intervention" OR "adaptive interface") AND (driver OR vehicle OR automotive OR "human-vehicle interaction")
{LLM term} + {reasoning/decision support term} + {driver assistance context}	("large language model" OR LLM OR GPT) AND ("reasoning chain" OR "decision support" OR "commonsense reasoning" OR "causal reasoning" OR "context-aware decision making") AND (driver OR ADAS OR DMS OR automotive)
